# Mass spectrometry imaging of mice brain lipid profile changes over time under high fat diet

**DOI:** 10.1038/s41598-021-97201-x

**Published:** 2021-10-04

**Authors:** Gianluca Sighinolfi, Samantha Clark, Landry Blanc, Daniela Cota, Boutayna Rhourri-Frih

**Affiliations:** 1 CBMN, Bordeaux, France; 2 Physiopathologie de la Plasticité Neuronale, U1215, Neurocentre Magendie, INSERM, 33000 Bordeaux, France; 3 Physiopathologie de la Plasticité Neuronale, U1215, Neurocentre Magendie, University of Bordeaux, 33000 Bordeaux, France

**Keywords:** Chemical biology, Biomarkers, Lipidomics, Lipids

## Abstract

Overweight and obesity have been shown to significantly affect brain structures and size. Obesity has been associated with cerebral atrophy, alteration of brain functions, including cognitive impairement, and psychiatric diseases such as depression. Given the importance of lipids in the structure of the brain, here, by using 47 mice fed a high fat diet (HFD) with 60% calories from fat (40% saturated fatty acids) and 20% calories from carbohydrates and age-matched control animals on a normal chow diet, we examined the effects of HFD and diet-induced obesity on the brain lipidome. Using a targeted liquid chromatography mass spectrometry analysis and a non-targeted mass spectrometry MALDI imaging approach, we show that the relative concentration of most lipids, in particular brain phospholipids, is modified by diet-induced obesity (+ 40%of body weight). Use of a non-targeted MALDI-MS imaging approach further allowed define cerebral regions of interest (ROI) involved in eating behavior and changes in their lipid profile. Principal component analysis (PCA) of the obese/chow lipidome revealed persistence of some of the changes in the brain lipidome of obese animals even after their switch to chow feeding and associated weight loss. Altogether, these data reveal that HFD feeding rapidly modifies the murine brain lipidome. Some of these HFD-induced changes persist even after weight loss, implying that some brain sequelae caused by diet-induced obesity are irreversible.

## Introduction

Diet-induced obesity is at present one of the most prevalent and costly diseases worldwide^1^, as this condition is often associated with type 2 diabetes, cardiovascular and neurodegenerative pathologies and several forms of cancer, resulting in a decline in both quality of life and life expectancy^2^. Obesity is substantially a life-style, environmentally-driven pathology, which results from the ingestion and storage of more calories than what the organism actually needs^3^. In mammals, energy homeostasis is finely regulated by the central nervous system (CNS). What we eat has a clear impact on brain function and an unbalanced diet with hypercaloric food rich in saturated fats and sugars is a risk factor for the development of brain diseases, including obesity^2^.

Lipid content represents more than half of the brain’s dry weight and plays a pivotal role in brain integrity. In particular, the brain is highly rich in complex lipids, such as glycerophospholipids, sphingolipids [sphingomyelin (SM), cerebrosides, ceramides (Cer) sulfatudes (SF) and gangliosides] and cholesterol^4^. The glycerophospholipids class includes 18 different categories, among them: phosphatidylethanolamines (PE), phosphatidylserines (PS), phosphatidylinositols (PI) and phosphatidylcholines (PCs)^5^. Phospholipids and sphingolipids play a critical role in the bilayer structure of cell membranes, since they allow the amphilicity of the membranes thanks to their polar head group and apolar chain. Within the brain, they also have a signaling role since PIs give rise to phosphorylated inophosphoinositides that act as second messenger^6^.

The interest in investigating the neuromodulatory role of lipids has therefore grown in the last decades and has been associated with pathological conditions.

For instance, Mendis et al. have described changes in the lipid composition of the hippocampus in Alzheimer’s disease^7^. Gonzalez et al. have demonstrated a depletion of different sulfatid lipid species in both gray and white matter areas of the frontal cortex in Alzheimer’s disease^8^. Other changes in the brain phospholipids and proteins were observed in animal models of depression^9^, while exposure to cocaine was associated with changes in phospholipids composition in the hippocampus and cerebellum^10^.

However, relatively little is known concerning brain lipid composition and its alteration in the context of obesity.

In particular, few studies have investigated the impact of the consumption of specific nutrients, such as sucrose, or of a high-fat diet (HFD) rich in saturated fats on brain lipid composition. These investigations focused on n-6 and n-3 fatty acid levels^11^, as changes in the balance of these fatty acids can directly affect neurotransmission and favor neuroinflammation^12,13^. In turn, these modifications have been associated with weight gain and obesity^14^. In rats fed a lard-enriched chow (high in saturated/low in polyunsaturated fatty acids) for eight weeks, hypothalamic insulin signaling and insulin-dependent regulation of food intake were drastically impaired, independently of the hypothalamic fatty acids compostion^15^.

A recent study using shotgun lipidomics has revealed an increase of amyloids plaquets under HFD in Alzheimer disease, and the modulation of 24 lipids subspecies with a specific alteration of cardiolipids in obese mice^16^ Other HFD-induced lipidome perturbations were described in the cortex, hippocampus, hypothalamus, and olfactory bulb of mice by using untargeted LC–MS–MS analysis, which showed an increase of all lipid species in all these brain structures^16^.

In these studies, brain fatty acid content was generally analyzed after several weeks of exposure to the HFD, when an obese phenotype has already developed, therefore not allowing distinguishing the impact of the diet on brain composition, before the development of obesity.

Other studies have shown correlations between circulating levels of LPCs, SM, nonesterified fatty acids (NEFAs), PCs and obesity^17^, suggesting that alterations in lipid metabolism play an important role in the disease.

In depth evaluation of brain lipidome could help understand underlying biological mechanisms for increased disease risk and consequently help determine specific therapeutic approaches.

Therefore, in the current study we have evaluated the impact of a HFD on brain lipid content in mice, by first assessing changes in brain composition after only 3 days of HFD consumption. We then evaluated brain lipid profile after 16 weeks of HFD, once that a clear obese phenotype was established, as well as during and after weight loss, by switching animals back from HFD to a control chow diet. In our lipid profile analysis we focused on phospholipids, such as phosphatidyl choline, phosphatidyl ethanolamine, phosphatidyl serine and phasphatidyl inositol. We performed these sets of analyses on the cortex, the striatum, the thalamus, the amygdala and the hypothalamus. These brain regions, and in particular the hypothalamus, the striatum and the cortex, are well known to play different and complementary roles in the regulation of feeding behavior, body weight and whole body energy metabolism^18–21^.

To ensure the specific identification and mapping of the brain lipidome, we have developed a unique approach that combines high resolution untargeted mass spectrometry imaging (MSI) and a targeted LC–MS/MS, enabling the analysis of specific structures of the brain in their native state and after obesity induction.

MSI is a powerful tool that allows untargeted investigations into the spatial distribution of molecular species in tissues. It has the capability to image thousands of molecules, such as metabolites, lipids, peptides, proteins, and glycans, in a single experiment without labeling^22^. The combination of information gained from MSI and LC–MS/MS analyses described here therefore provides a comprehensive database of the brain lipidome and of its spatial organization in relation to different exposure times to HFD, to the development of obesity and to its reversal.

## Results

### Body weight evolution

**Figure 1:**
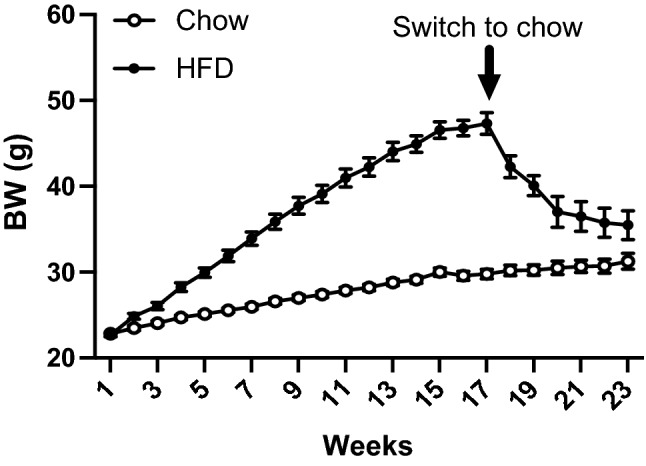
Time course of the study—we used in total 47 mice Data are mean ± SEM.

**Figure 2:**
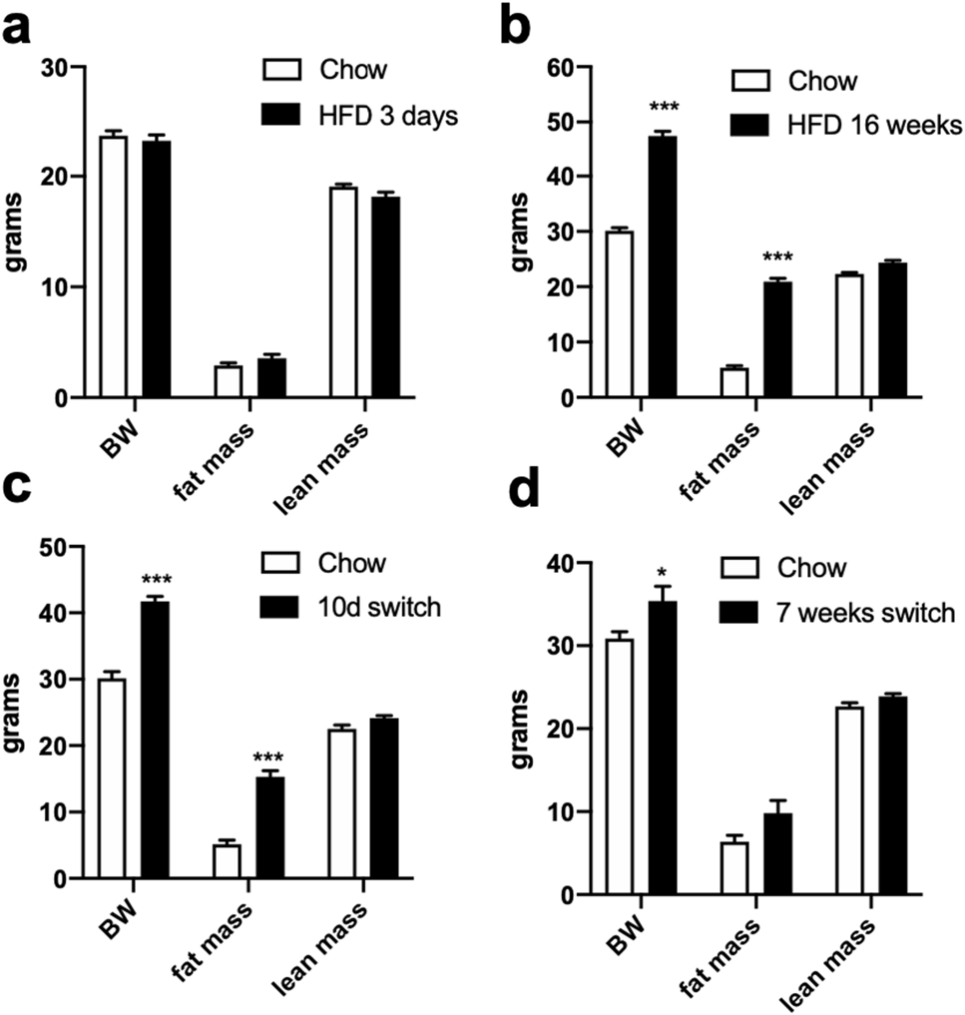
**(a)** 3 days HFD (6 mice/group); **(b)** 16 weeks HFD (17–18 mice/group), **(c)** 10 days reversal (5–6 mice/group); **(d)** 7 weeks reversal (6 mice/group); data are mean ± SEM. *p < 0.05, ***p < 0.0001.

Figure 1 illustrates the changes over time in the body weight (BW) of the mice used for the study. After 3 days on HFD, mice did not show any difference in the BW, fat or lean mass as compared to chow-fed controls (Fig. 2a, T0). After 16 weeks on HFD, mice had clearly become obese, with a significant increase in BW and fat mass (Fig. 2b, T16), as expected. After 10 days of diet switch, previously HFD-fed mice switched to chow were still frankly obese, with a much greater BW and fat mass than chow-fed controls (Fig. 2c,T18). Finally, BW and fat mass decreased in previously HFD-fed mice after 7 weeks of chow, with a fat mass that was no longer significantly different from the one of chow-fed controls (Fig. 2d, T23).

### LC–MS–MS results

#### Principal component analysis

Using LC–MS–MS results of three brains from HFD-fed or diet reversed animals and three brains from age-matched chow-fed controls, a PCA was performed on the log ratio HFD-fed (or reversed)/ chow-fed control at the four time points of the study (T0, T16, T18 and T23).

**Figure 3:**
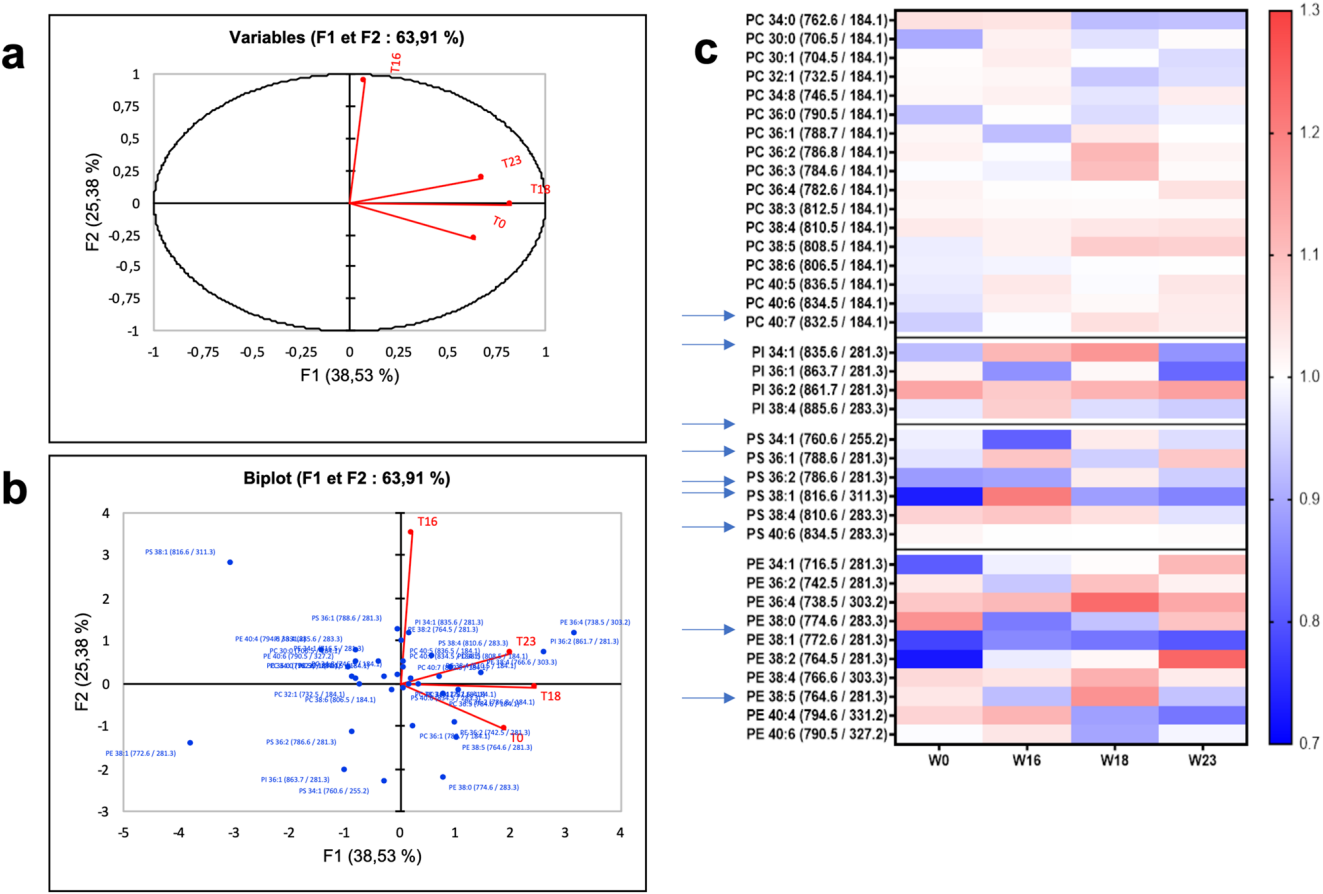
**(a)** Loading plot of principal component analysis of variables with F1 representing 38.53% of variabiliting and F2 25.38% based on the LC–MS–MS analysis in positive and negative mode of 24 mice (12 under high-fta diet and 12 control) at four stages (T0, T16, T18 and T22w). **(b)** PCA score plot of log ratio (obese/chow) using XLSTAT software (Addinsoft 2020. XLSTAT statistical and data analysis solution. New York, USA). **(c)** Heat map analysis of obese/control ratio based on the LCMS–MS quantification of phospholipids showed significantly modified lipids intensity over time (Graph Pad Prism V8).

Table1 represents the eigenvalues and the percentage of variance explained by each principal component, as well as the accumulated variance. The first three components are needed to explain 85.5% of the total variability in the phospholipids of the brain section. The first component (F1) explains 38.53% and 25.38% is explained by the second component (F2), together accounting for 63.91% of the total variation (Table1). The percentage of the accumulated variance in the first factor is justified by the nature and quantity of the variables evaluated. So even with the standardization of the data, four components were necessary to reach the adopted selection criteria (≥ 1.0), with 100% of the accumulated variance. Most of the evaluated phospholipids (63.91%) made significant contributions to the total variation of the data (Fig. 3b).

**Table 1:** Principal components (PC), eigenvalues, percentage of the variance for each component and the total variance of brain lipids in HFD-fed and Chow-fed mice from LC–MS–MS data.

	F1	F2	F3	F4
Eigenvalues	1.541	1.015	0.867	0.576
Variance (%)	38.532	25.380	21.677	14.411
Variance accumulated (%)	38.532	63.912	85.589	100.000

The loading plot of variables (Fig. 3a) shows that PC2 is mostly explained by the variable at T16 while T0, T18 and T23 constituted the PC2. The biplot depicts phospholipids whose variation explain group clustering, distinguishing T16 from the other time points (Fig. 3b). A heat map based on the mean ratio of phospholipids in HFD or reversed mice and chow-fed control mice at different time points (T0, T16, T18 and T23) showed clearly the phospholipids that significantly vary at T16 (Fig. 3c).

Among the 37 phospholipids identified in brain sections using LC–MS–MS, 17 PCs, 4 PI, 6 PS and 10 PE were identified.

#### Differentially expressed lipids in positive mode

The majority of lipids observed in positive mode in LC–MS–MS are PCs. Among a list of 66 PCs, 17 were identified and quantified.

**Figure 4:**
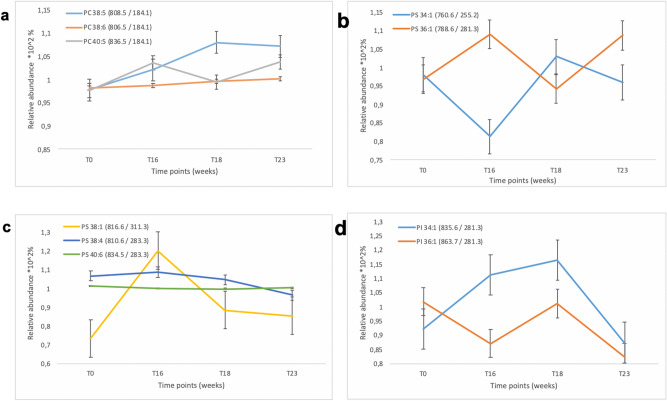
Relative variation of log ratios of peaks intensities obese on control over time: **(a)** Phosphatidyl choline variation (PC 38:5; PC 38:6 and PC 40:5). **(b,c)** Phosphatidyl serin variation (PS 34:1; PS 36:1; PS 38:1; PS 38:4 and PS 40:6). **(d)** Phosphatidyl Inositol variations (PI 34:1; PI 36:1).

The analysis did not show a significant difference across weeks, since only 2 of the 17 identified PCs had a difference upwards or downwards (Fig. 4a).

The normalized relative intensities of PC 40:5 was found to increase over time and with weight gain. Then it went down at T18, during weight loss and back up at T23, after 7 weeks of switch to chow. Conversely, the normalized relative intensity of PC 38:5 increased continuously, even in response to the chow switch at T18 and remained unchanged 7 weeks after from the diet switch.

#### Differentially expressed lipids in negative mode

The quantitative LC–MS–MS analysis showed variation in the concentrations of several negatively charged phospholipids (PS, PI and PE). Relative concentration ratios between brains from HFD-fed mice and chow-fed controls over time revealed negatively correlated phosphatidylserines, like PS 36:1, which was found to vary inversely with PS 34:1 at T16, T18 and T23 (Fig. 4b). Whilst, PS 38:1 increased at week 16 and went back to baseline with the chow switch (Fig. 4c). On the other hand, PS 40:6 and PS 38:4 remained unchanged over the course of the study (Fig. 4c).

For the phosphatidylinositols, the proportions of PI 36:1 and PI 34:1 varied inversely, with the PI 34:1 ratio increased at T16 and T18 and decreased at T23, while the PI 36:1 decreased at T16, went back to baseline at T18 and decreased again at T23 (Fig. 4d).

### MSI results

#### Brain segmentation with MSI

**Figure 5:**
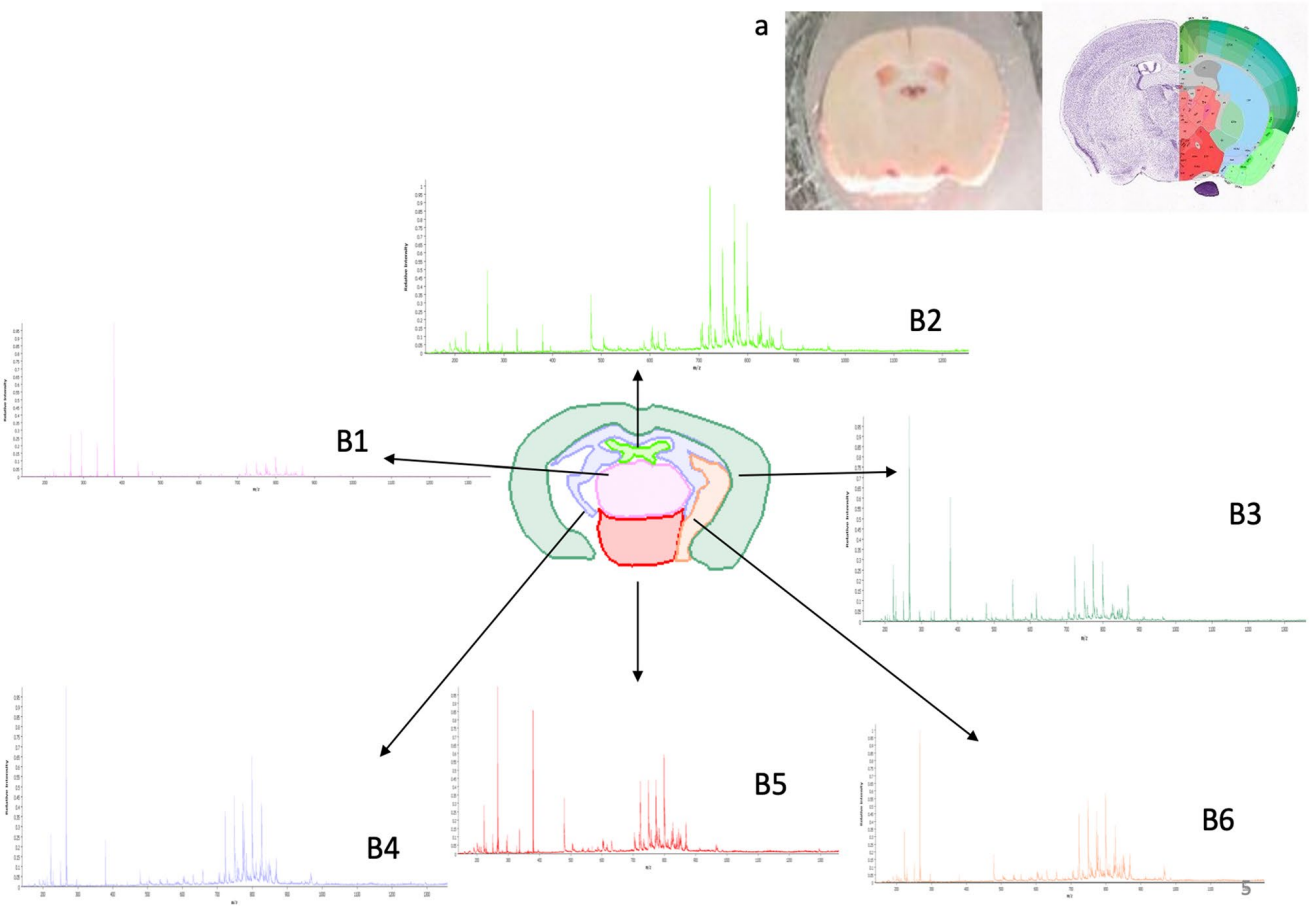
**(a)** Cortical brain section at 5.9 mm depth from Allen Brain. **(b)** Edges of the cortex, the striatum and the hypothalamus, thalamus, amygdale and ventricles drawn with Scills and FlexImaging 4.1 software (Bruker Daltonics Inc.). B 1–6 mean spectra of brain regions in negative mode in the range mass of *m/z* 200–1400, normalized with the TIC. (b1) Thalamus, (b2) ventricles, (b3) cortex, (b4) amygdale, (b5) hypothalamus and (b6) striatum.

Using the MALDI–TOF–TOF at low resolution and a specific software (SCiLS, Bruker), we draw the edges of the cortex, striatum, hypothalamus, thalamus, amygdala and of the brain ventricles guided by the Allen mouse brain atlas (Fig. 5a). Serial sections from the brain of a chow-fed control mouse in positive and negative mode were analyzed and a normalized average mass spectrum was generated for each region (Fig. 5b1–6). We therefore observed a regional distribution of lipids with higher relative intensity of lysophospholipids (Lyso) (200–400 *m/z*) in the cortex and hypothalamus (Fig. 5b3 and b5). While, hypocampus and choroid plexus (Fig. 5b2, b4 and b6) had higher relative intensity of phospholipids (PL) (500–900 *m/z*). In contrast, in the thalamus, the normalized average spectrum had low relative intensity of both Lyso and PL (Fig. 5b1).

**Figure 6:**
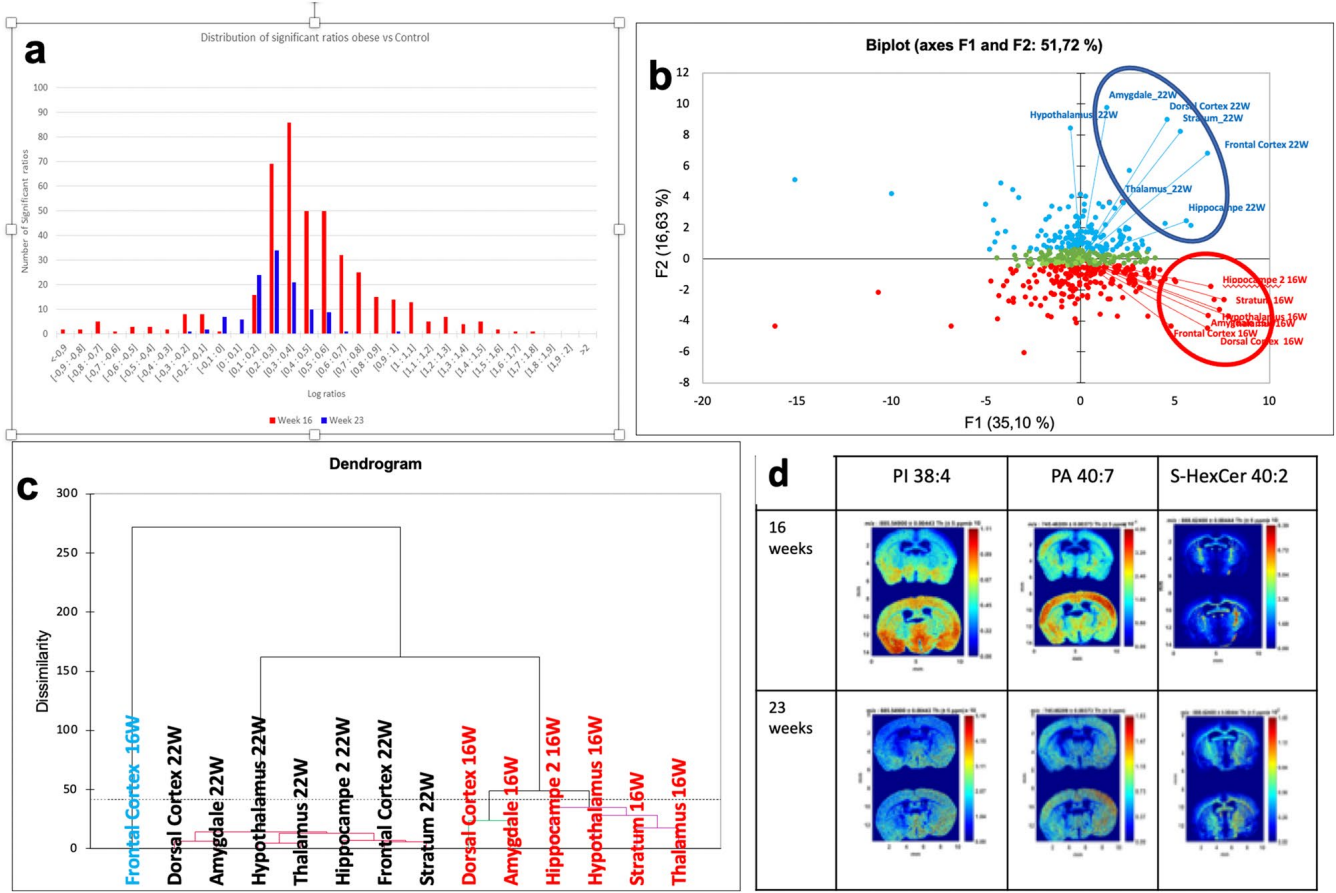
**(a)** Histogram of log ratios of lipids at 16 weeks (red square) and 23 weeks (blue square), data resulting from MALDI–MSI spectrum in negative mode. **(b)** PCA score plot of log ratios of lipids signal of in obese and control brain sections at week 16 and week 23, blues dots representing lipids positively correlated with 16 weeks stage, blue dots are lipids correlated with 23 weeks stage, green dots are persistent lipids at both stages (16 & 23w). **(c)** Ascendant hierarchical clustering of brain regions at 16 weeks (red color) and 23 weeks (blue color) based on the log ratio of lipids detected and identified in the brain using XLSTAT software (Addinsoft 2020. XLSTAT statistical and data analysis solution. New York, USA), **(d)** MALDI Images generated of *m/z* found significantly differentiated in obese brain (the bottom image) in regard to control (the top image) (p < 0.01): phosphatidyl inositol PI 38:4, phosphatidic Acid (PA 40:7) and S-HexCer 40:2 (MSi Reader V1.01).

The MSI screening method was then used to obtain the anatomical distribution patterns of lipids in coded images and confirmed the regional specificity of the brain lipids distribution. After this initial analysis, we compared the different brain regions using MALDI-IMS high resolution in order to identify brain lipids changes occurring in response to chronic HFD exposure (T16) as well as after long-term diet switch and body weight loss stabilization (T23).

#### Global lipid profile

Using MALDI-IMS in negative mode on brain samples, a total of 650 lipids were identified but only 430 lipids were kept after Metaspace FDR. Among these, 216 lipids at W16 and 116 at W23 were identified with Lipid Maps. Of note, we did not observe significant differences in positive mode in LC–MS–MS and in MALDI–TOF–TOF (data not shown).

Simultaneous analysis of brain sections from mice fed with HFD (or reversed) and age-matched chow-controls was carried out, with biological triplicates for each. Only lipids with a signal greater than 1% of the internal standard signal were kept for interpretation.

The signal intensities obtained for each identified lipid were systematically normalized to the signal from the internal standard sprayed with the matrix.

After normalization, as done before, we calculated the ratio of the HFD (or reversed) signal over the chow control signal and the average of the ratios was obtained for each individual lipid.

The distribution of the log ratios at T16 and T23 is represented in Fig. 6A. Log ratios at T16 were much higher than at T23, with the number of lipids with a log ratio centered on the value 0.4 at T16, while the distribution of the log ratio at T23 was centered around 0.3, with lipids’ abundance higher at T16 than at T23. This overall means that a major proportion of lipids from brains of mice fed a HFD for 16 weeks had concentrations 2 times higher than in the brain sections from chow-fed controls. While at T23, after diet switch, only very few lipids were still increased as compared to the chow-control group. A PCA was carried out to examine the correlation between the lipids’ concentrations at T16 and T23 in Fig. 6b. By choosing components 1 & 2 we were able to describe 51.72% of the variability of lipids within brain regions and time points. This analysis revealed specific distribution of lipids in different brain regions depending on the two conditions (T16 and T23) analyzed.

In particular, and despite an apparent grouping of lipids around the center of the biplot (green dots), testifying the presence of unchanged lipids between T16 and T23, we were able to distinguish two major groups of lipids. The first group was located on the upper part of the second component (F2), which correlated with T23, and the second group was located in the second lower part of axis 2, and was related to T16. In each group of lipids we noticed a subdivision into two subgroups according to axis 1 of the biplot, thus marking the presence of subgroups of lipids positively and negatively correlated and with the experimental condition.

Considering the low percentage of variability that was described by the PCA (51.72%), we decided to perform an agglomerative hierarchical clustering (AHC), which has the advantage of describing the total variability in the chosen observations (Fig. 6c).

To facilitate the clustering interpretation, we preferred to transpose data before the AHC processing. The results described in the dendrogram confirmed the general trend with different regions of the brain at T16 that showed lower Euclidean distance and were grouped together in one class. This stems from the presence of a large common class of lipids characteristic of the obese state at T16, independently of the brain region studied. The only exception that emerged from this clustering was the frontal cortex at T16, which represented itself an individual class with a very important Euclidean distance from the remaining regions at T16.

Different brain regions at T23 were also grouped in a single class, indicating the presence of the same families of lipids in these brain structures after diet switch and weight loss.

We then illustrated in Fig. 6d three examples of overexpressed lipids at T16 in an obese brain (section at the bottom) as compared to the chow control (section at the top). Specifically, we observed an overexpression of phosphatidyl Inositol (PI 38:4), in the cortex and the hypothalamus, of PA 40:7 in the frontal cortex and of the sulfated hexose ceramide 40:2 in the thalamus of the obese brain relative to the chow controlAll these changes were normalized at T23.

#### Comparison of lipid profile over time in the cortex

**Figure 7:**
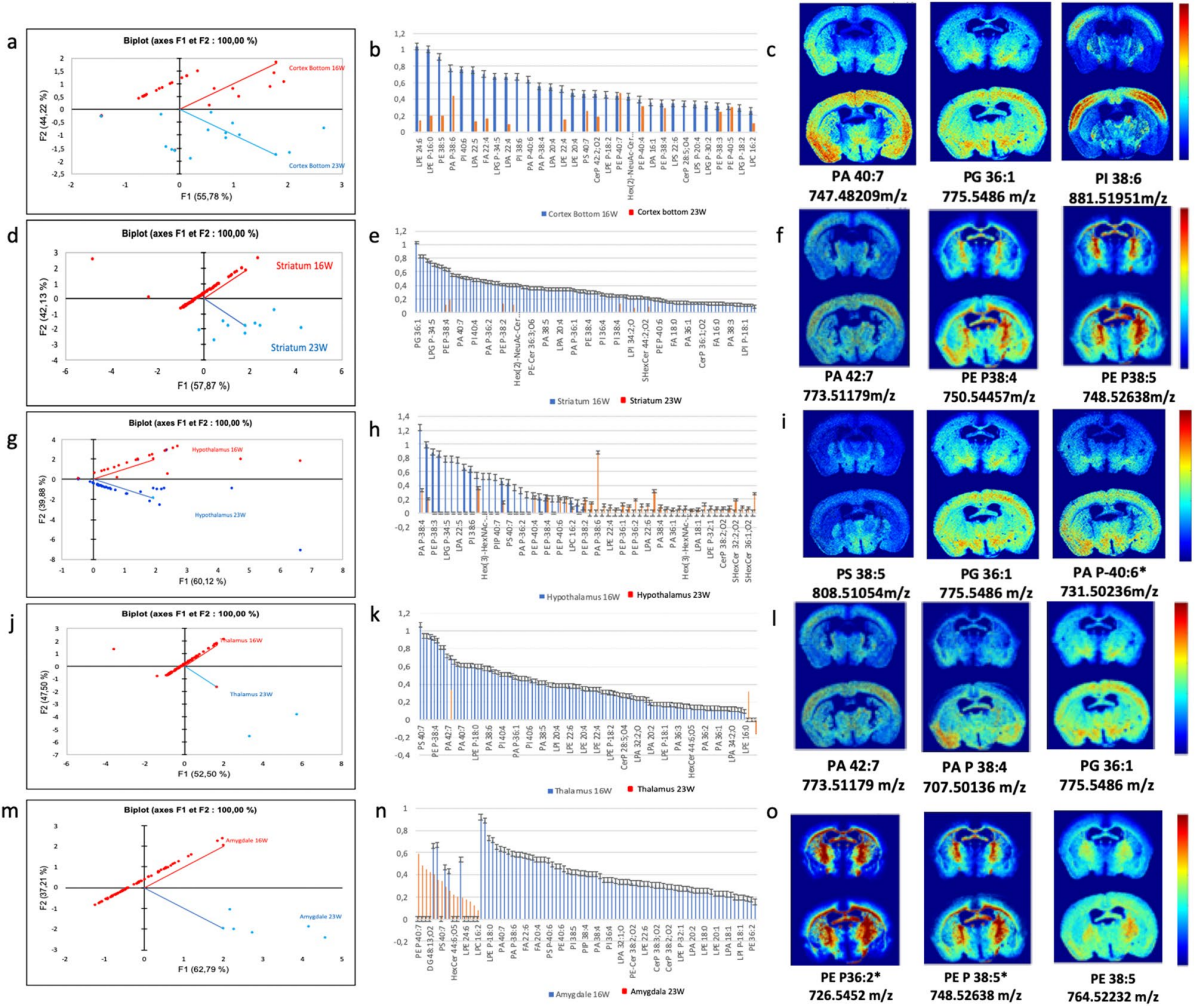
**(a)** PCA score plot of log ratios of lipids signal of cortex region in obese and control brain sections at week 16 and week 23, **(b)** histogram of log ratio obese vs control in the cortex, **(c)** MALDI images generated of *m/z* found significantly differentiated in obese brain in regard to control (p < 0.05) in the cortex region. **(d)** PCA score plot of log ratios of lipids signal of striatum region in obese and control brain sections at week 16 and week 22, **(e)** histogram of log ratio obese vs control in the striatum, **(f)** Images generated of *m/z* found significantly differentiated in obese brain in regard to control (p < 0.05) in the striatum region. **(g)** PCA score plot of log ratios of lipids signal of hypothalamus region in obese and control brain sections at week 16 and week 23, **(h)** histogram of log ratio obese vs control in the hypothalamus, **(i)** images generated of *m/z* found significantly differentiated in obese brain in regard to control (p < 0.05) in the hypothalamus region. **(j)** PCA score plot of log ratios of lipids signal of thalamus region in obese and control brain sections at week 16 and week 23. **(k)** Histogram of log ratio obese vs control in the thalamus, **(l)** images generated of *m/z* found significantly differentiated in obese brain(the bottom image) in regard to control (the top image) (p < 0.05) in the thalamus region. **(m)** PCA score plot of log ratios of lipids signal of amygdala region in obese and control brain sections at week 16 and week 23, **(n)** histogram of log ratio obese vs control in the amygdala, **(o)** images generated of *m/z* found significantly differentiated in obese brain in regard to control (p < 0.05) in the amygdala region (PCA were obtained using XLSTAT software (Addinsoft 2020. XLSTAT statistical and data analysis solution. New York, USA and mass spectrometry images with MSi reader V1.01).

Among the 216 lipids initially identified, only 44 were found to significantly differ in the cortex (p < 0.05) and were used for the PCA (Fig. 7a). 30 lipids (in red) were specific of T16, testifying for high relative concentration of this group of lipids in the obese brain. Differently, 14 lipids were associated with T23 and therefore typical of the diet reversal phase.

As done before, we calculated the log of ratios of obese (or reversed) intensity vs chow-control intensity for each individual identified lipid and used it to classify lipids occurrence in the obese (or reversed) brain, from higher to lower occurring ones (Fig. 7b). Most lipids were upregulated at T16 as compared to T23, with lipids intensities in the obese state varying from 1.5 to 10 times the lipid intensity at T23. Lyso-phospholipids (Lyso-phosphatidyl ethanolamine PE 24:6) were among the most overexpressed lipids, reaching a relative concentration 10 times higher in the obese brain at T16 (Fig. 7b).

Figure 7c shows the MSI of brain sections at T16 illustrating the distribution and expression of three lipids found overexpressed in the cortex (highlighted area). The images clearly show, as for the graphs, an overexpression of these lipids in the cortex (as defined in Fig. 5b) in the obese state (bottom images) as compared to the chow-control condition (top images).

#### Comparison of lipid profile over time in the striatum

As for the cortex, most of the lipids were overexpressed at T16 in the striatum as well (red dots, Fig. 7d). On the right down quartile of the biplot a few lipids correlated with diet reversion at T23. The log ratio histogram showed specific lipids overexpressed in the striatum in the obese state at T16 (Fig. 7e), with phosphatidyl glycerol PE 36:1 being 8 times higher in the obese than in the chow control.

Figure 7f shows an example of images with over expression of lipids (especially plasmalogens PE P 38:5) in the striatum region of obese animals at T16.

#### Comparison of the lipid profile over time in the hypothalamus

Similarly to the cortex and striatum, also in the hypothalamus the majority of phospholipids were overexpressed at T16 as compared to chow-fed control. The principal component analysis showed a symmetric dispersion of lipids according to the first axis F1, which represents 60.12% of the total variability. Half of the points were associated with obesity at T16, while the other half correlated positively with the diet switch at T23, revealing the persistence of high abundance of lipids after switch to chow and weight loss (Fig. 7g).

The histogram of log ratios showed 26 phospholipids with ratio higher than 2 at T16. Among those lipids, phospholipids PA 38:4 and PA 38:6, PG 36:1 and PG38:4, and various lyso-phospholipids, plasmalogens and ceramides were found highly expressed in the hypothalamus from obese mice (Fig. 7h). 26 other lipids were highly expressed after the switch to chow and weight loss. These lipids were mainly plasmalogens, also called ether lipids. The following were already increased with weight gain at T16 and their ratios was even higher at T23: PA P-38:6; PA P-40:6; PAP-38:4; PE P-38:5; PI P-38:5; PE P-40:4; PE P-38:4; PE P-40:5; PE P-40:6; PE P-38:2; PE P-36:2. These are ethers of phosphatidyl ethanol amine, phosphatidyl ethanol or phosphatidyl inositol, all with fatty acyl chain length from 36 to 40 and number of unsaturation from 2 to 6.

Figure 7i shows representative MALDI images of lipids with log ratio > 0.5 at T16 and we have indicated with an (*) those lipids that remained high at T23, after chow switch. Thus, even after the diet switch and body weight loss, the hypothalamus, a structure that critically controls food intake and body weight (16, 17, 19), clearly shows permanent changes in lipid composition.

##### Comparison of lipid profile over time in the thalamus

The PCA for the thalamus showed that all the lipids except 3 were aligned and correlated at T16 (Fig. 7j).

Log-ratio analysis showed that the lipid with the highest ratio at T16 in this brain region was the phosphatidyl serine PS 40:7 (Fig. 7k). The thalamus had high presence of several poly lipids with a fairly long medium chain length (PG 36:1; PA 42:7; PI 38:6; PA 40:7). As with the previous brain regions, we found abundance of ether lipids (PE P-38:3;PE P-38:4; LPG P-34:5; LPE P-16:0; PA P-38:4; PA P-40:6; PE P-38:2; PA P-36:2; PE P-36:2; PE P-38:5) and lyso-phospholipids (LPA 22:5; LPA 22:6; LPA 22:4) at T16. Figure 7l shows representative images of lipids whose log-ratio was the highest at T16.

##### Comparison of lipid profile over time in the amygdala

The PCA describing the distribution of lipids in the amygdala and their correlation with experimental conditions of T16 and T23 showed results similar to those already described for the thalamus, with an apparent alignment of lipids correlated with the obese state and very few lipids related to the diet switch at T23, which however were highly present (Fig. 7m). By calculating log-ratios (Fig. 7n), we found that the majority of lipids increased in the obese state at T16, were normalized at T23.However, 5 lipids that were high at T16 aremained high at T23, and were called “persistent lipids” (PE P-38:5; PE P-36:2; PE P-36:1; PI 40:6 and PI 38:6). Finally there were also lipids that did not have a high ratio at T16, but that were increased at T23 (PE P-40:7; PE P-38:2; PE P-38:1; PG 36:1; PS 40:7; HexCer 44:6;O5; S-HexCer 42:2;O2; LPE 24:6; CerP 44:6;O6; CerP 42:2;O2; LPC 16:2).

Figure 7o shows representative images by MALDI-IMS at T16 illustrating the spatial distribution of lipids in the amygdala and their more abundant relative intensity in the obese brain as compared to chow-control. We identified with an (*) those lipids that were still highly present at T23, after diet switch and weight loss.

**Figure 8:**
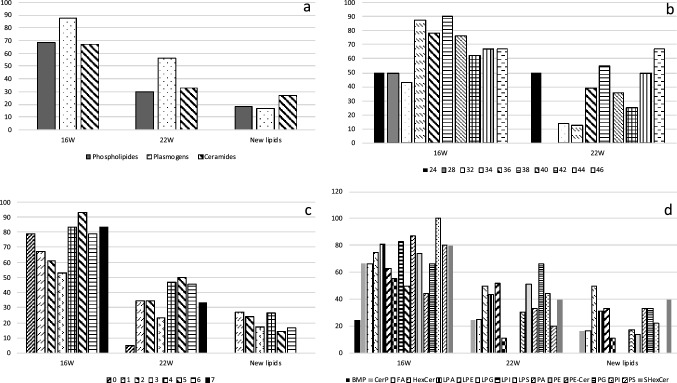
Lipid’s relative abundance variations sorted by **(a)** the lipid’s type (phospholipids, ether lipids and ceramides), **(b)** the carbon chain length, **(c)** the number of unsaturation and **(d)** lipid species at 16 and 23 weeks.

Overall the analyzed brain areas, we classified the identified lipids in three species (phospholipids, ceramides and plasmogens) and compared their variation between T16 and T23 (Fig. 8a). This analysis revealed that plasmogen relative concentrations are the most modified, with a strong increase in the obese state, and remain high after weight loss. We also analyzed the evolution of lipid structure in term of carbon chain length and number of unsaturation. Persistent lipids after diet switch were clearly those with a high number of unsaturation and long carbon chain (Fig. 8b,c).

## Discussion

This study demonstrates that feeding mice with a HFD leading to obesity results in lipid profile changes and that these changes are localized in the specific brain region investigated. Importantly, some changes in the brain lipidome were still present 7 weeks after switch back obese animals to chow and observing weight loss (which by week 23 had plateaued, see also Fig. 1). This evidence implies that these persistent changes in lipid composition are not merely a direct effect of the diet composition, but could indicate a definitive alteration in the structure of certain areas of the brain due to a previous episode of obesity. This was particularly true for the cortex, the striatum and the hypothalamus, brain regions critically involved in the regulation of different aspects of feeding behavior, of body weight and of peripheral metabolic responses (Supplementary Fig. S1).

The results obtained in LC–MSMS showed very little variation of the lipid profile in positive mode which mainly concerned PC 38: 5 and PC 40: 5. These lipids had shown a slight variation between obese mice and controls at T16 (Fig. 4a). Whilst, in the LC–MSMS analysis in negative mode, two phospholipids species were found varying with weight gain: the phosphatidylserine mono unsaturated (PS 36:1 and PS 38:1), and the phosphatidyl inositol (PI 34:1 and PI 36:1). The LC–MSMS analysis in negative mode also revealed a recovery of the initial ratios of lipid signals after 7 weeks of switch to chow for the majority of lipids. Nevertheless the presence of certain types of lipids persisted even after weight loss. These observations led us to focus on the IMS analysis of brain sections in negative mode, which revealed several lipids varying significantly in association with the obese state and within specific brain regions.

The general conclusion from the IMS studies we have conducted, is that all lipids are overexpressed in diet-induced obese mice as compared to age-matched chow-fed controls at T16, and in particular the plasmalogens or ether lipids. These phospholipids are a subclass of glycerophospholipids (GP) that have an alkyl chain attached by an ether bond at the sn-1 position of the glycerol backbone^23^.

They also represent the major part of the persistent modifications after weight loss (Fig. 8a).

Overall, these findings provide evidence for an ether lipid signature in the brain of diet-induced obese mice. This signature is characterized by an increased content of alkenyl forms of phosphatidylethanolamine (GPEtn).

In addition, these lipid species have an increased relative abundance of a longer carbon chain length (> 36C) (Fig. 8b) with a higher number of double bonds (> 4 insaturations) (Fig. 8c).

Of note, Plasmalogens constitute ~ 15–20% of total phospholipids in cell membranes, with ≥ 50% of the GPEtn fraction in brain, heart, neutrophils and eosinophils^24^. Farooqui et al., have demonstated that plasmalogen content of tissues generally decreases in aged mammals^25^. It was also showen a low lipoxidative damage in long-lived animal species, with an accumulation rate that inversely correlates with longevity^26^.

Plasmalogens derivatives from phosphatidylethanolamine species with long carbon chains and with a high number of double bonds were also found reduced in the plasma of centenarians compared with young people^27^.

Given the above findings, we may hypotehsize that positive correlation between PE Plasmogens derivatives and obesity will likely correlate with the reduced life expectancy observed among obese subjects^28,29^.

The second species of lipids which was overexpressed in obese mice at T16 were lipids of the ceramides family, which remained expressed even at T23, after the switch to chow and weight loss, with in addition the appearance of new ceramides not previously modified in the brain of obese animals. This is not at all surprising given the role that ceramides play as metabolic intermediaries in nutrition, weight gain and obesity^30^. In particular, ceramides accumulation in the brain due to pharmacology or genetic manipulation directly alters food intake, energy expenditure and glucose homeostasis^30^. Here (Fig. 8d), we were able to distinguish two subclasses of ceramides which were found to be overexpressed in the brain of obese animals. These were the ether ceramides and the hexose ceramides, but only the hexose ceramides were still high after the switch to chow and the weight loss at T23.

A recent lipid-gene regulatory network study has also revealed coregulations of triacylglycerol with phosphatidylinositol/lysophosphatidylinositol and with hexosyl-ceramide^31^, which may explain the high level of hexosyl-ceramide that we have observed after weight loss.

Another interesting finding was that the expression of Bis(monoacylglycero)phosphate (BMP, and specifically BMP 18:0) was increased at T16 and, although decreased at T23 after weight loss, still remained high as compared to age-matched chow-fed controls (Fig. 8d).

BMP is a negatively charged glycerophospholipid with an unusual sn-1;sn-1’ structural configuration. BMP is thought to play a role in glycosphingolipid degradation and cholesterol transport^32^. Elevated levels of BMP have been found in diseases associated with lysosomal storage material. Interestingly, BMP content in brain of humans and animals with either GM1 or GM2 ganglioside storage diseases is significantly greater as compared to brains of normal subjects^33^. Another peculiarity of BMP is its specific biophysical and functional properties confered by the incorporation of docosahexaenoic acid (DHA) compared to other polyunsaturated fatty acids^34^.

This can explain its presence only in the HFD condition at T16, leading to the assumption that its role is directly linked to food metabolism^35^.

We have investigated the impact of HFD, obesity development and its reversal by dietary intervention on the brain lipidome, clearly showing that the type of diet eaten impacts the brain lipids profiles. The novel aspect of this work is that it demonstrated, using IMS, the variation in lipids distribution and concentration within different regions of the brain, such as the cortex, the striatum and the hypothalamus, which are known to play critical roles in the regulation of feeding behavior and whole-body energy metabolism^18–21^. It was concluded that numerous lipids vary subsequently to chronic exposure to HFD, especially plasmalogens, ceramides and lyso-phospholipids, which were found to increase significantly in the obese state. The most surprising finding was the presence of a large number of lipids which, despite the switch back to chow and the consequent body weight loss, did not recover their initial relative intensity as compared to age-matched chow-fed controls, indicating the production of irreversible changes caused by chronic exposure to HFD and weight gain.

## Materials and methods

### Animal experimental procedure

Experiments were performed following the European Union recommendations (2010/63/EU) after approval from the French Ministry of Agriculture and Fisheries (authorization no. 3309004) and the local ethical committee of the University of Bordeaux and in accordance with the The ARRIVE guidelines 2.0.

Adult 7-week-old male C57bl6/J mice (Janvier Labs, Le Genest-Saint-Isles, France) were individually housed at 22 °C with a 12 h light–dark cycle (light off at 1 pm). Animals had free access to water and chow (13.5% of calories from fat, 61% of calories from carbohydrates, Standard Rodent Diet A03, SAFE, France) unless stated otherwise. Mice were acclimated for one week before the start of the study. At the start of the study, mice were randomly distributed in two groups and either maintained on chow for the length of the study or fed a HFD with 60% calories from fat (40% saturated fatty acids) and 20% calories from carbohydrates (D12492 Research Diets, New Brunswick, NJ) for 3 days (T0), or 16 weeks (T16). In order to study the possible reversal of brain lipid changes induced by HFD or obesity, 16 weeks HFD-fed mice were then placed back on chow and animals were analyzed after 10 days on chow (T18), or after 7 weeks on chow (T23) and compared to age-matched chow-fed control animals never exposed to HFD (See also Fig. 1 for the evolution of body weight). Changes in body weight were recorded weekly for the length of the study. At T0, T16, T18 and T23, 6 mice per group were killed by cervical dislocation and brains rapidly removed and fresh frozen for further analysis. In total 47 mice were used to generate data on body weight and body composition at the different time points; while brain analysis was conducted on three mice per group/time point chosen randomly and with technical repetition for each brain section.

### Body composition analysis

A nuclear echo magnetic resonance imaging whole-body composition analyzer (Echo MRI 900; EchoMedical Systems, Houston, TX, USA) was used^35^ to repeatedly assess body fat and lean mass changes in conscious mice during the study at T0, T16, T18 and T23 (Fig. 2).

### Chemicals

α-Cyano-4-hydroxycinnamic acid (HCCA), 1,5-Diaminonaphthalene (DAN) were supplied by Sigma-Aldrich. Trifluoroacetic acid (TFA), HPLC grade ethanol, Acetone, acetonitrile and isopropanol were supplied by Fischer Scientific UK.

### Extraction protocols

A two-step process was used for the extraction of PLs^36^. Briefly, sections adjacent to those used for imaging were removed and introduced into an eppendorf tube to perform lipid extraction. Brains sections were homogenized with 200 µL of 180 mM ammonium acetate, pH 8. The lysate was extracted with 990 µL of chloroform/methanol 17/1 (v/v) to eliminate apolar lipids. Then, the aqueous phase was re-extracted with 990 µL of chloroform/methanol 2/1 (v/v) to isolate polar lipids in the organic phase; this PLs extract was dried under vacuum (Speedvac, Thermo).

### LC–MS and LC–MS–MS methods

A Shimadzu gradient LC system (CBM-20A) interfaced to a QTrap mass spectrometer equipped with a TurboV electrospray source (SCIEX API 5500) was used for LC–ESI–MS/MS analysis of PLs. The separation of lipids was carried out at 40 °C on a C8 Synergi Fusion-RP 150 × 1 mm column, with 80 Å pore size, 4 µm particles (Phenomenex). Eluent A was isopropanol/methanol/H_2_O + 0.2% formic acid + 0.028% NH4OH; Eluent B was isopropanol + 0.2% formic acid + 0.028% NH4OH. The gradient elution program was: 0 min, 30% B; 5 min, 50% B; 30 min, 80% B; 31–41 min, 95% B; 42–47 min, 5% B; 52–62 min, 30% B. The flow rate was 40 µL/min^36^.

PLs extracts were dissolved in 400 µL of solvent B used for the reversed phase liquid chromatography analysis; 5 µL sample volumes were injected. The mass spectrometer was operated over a mass range of *m/z* 250–1100 at a 1000 Da/s scan speed. The ion spray voltage was set at 5500 V in the positive ion mode and 4500 V in the negative ion mode. The trap fill-time was set at 3 ms in the positive ion mode, and 5 ms in the negative ion mode.

A simple mass analysis was performed in both positive and negative mode to predict phospholipids identification following their retention time and *m/z* values.

Afterwards, we completed a parent ion scan. The combination of retention time and fragments identification based on the loss of the phospholipids head followed by subsequent loss of Sn2 and Sn1 fatty acid chains, allowed the final confirmation of the phospholipids. Finally the confirmation step consists in matching two fragments with a common precursor ion at the corresponding retention time.

The Multiple Reactions Monitoring (MRM) mode was used for the quantification step, where each phospholipid was distinguished by selecting the *m/z* of the precursor ion and major fragment ion. Before the quantitative analysis, 6 standards phospholipids were added to sample solution (PA 34:0; PS 34:0; PG 34:0; PC 26:0; PE 34:0; PI 31:1), and used respectively as internal standard to quantify different detected phospholipids.

After normalization with the internal standard signal, the relative abundance of each phospholipid species was calculated, for instance, a phosphatidyl choline relative abundance was calculated considering the ratio: concentration of the given PC to the sum of all PCs that are present in the sample.

$${\rm iPC X:y}\,\%\,=\,{\rm (iPC X:y/PC sum)} \times100.$$

The calculation of concentration, and the relative abundance of each lipid was performed in three different biological samples. The results were used to confirm if there was quantitative changes in brain lipids with weight gain.

### Tissue preparation for MSI

Mouse brains were frozen in a dry ice chilled isopentane and stored at − 80 °C. Frozen organs were transported from the − 80 °C freezer to a cryostat (Lyca, CM1950) on dry ice. For mounting, tissues were held atop a drop of HPLC grade water on the cutting stage and placed into the cryostat chamber, held at − 19 °C to equilibrate. Frozen brains were sliced into 12 µm sections. A mouse brain atlas was used to guide the experimenter in the collection of the samples.

The brain sections were directly deposited on indium tin oxide (ITO) coated conductive glass slides for MALDI imaging and dried and preserved in vacuum dissicator to remove moisture before matrix coating.

### Matrix application on brain sections

ITO-coated slides bearing mouse brain sections were scanned (HP Scanjet G4050) prior to matrix deposition. α-Cyano-4-hydroxycinnamic acid (HCCA) was used at concentration of 7 mg/ml in acetone/water, 0.2% TFA 1:1 (v:v) as a MALDI matrix for the analysis in positive ion mode and DAN was used for the analysis at 5 mg/ml in Acetone/water 7:3, supplemented with 16:0-d30-18:1 PA at 10 μg/ml as internal standard for normalization and Palmitate-d30 at 10 μg/ml for LockMass. Matrix was spray-coated using a homemade based on a glass Type A Meinhard nebulizer (Golden, CO)^37^. Matrix was applied in multiple cycles (spray generation, matrix incubation on the tissue section, and drying the sample using a soft nitrogen flow) to reach an adequate thickness without lateral delocalization of analytes (approximatively 1 ml matrix solution per slide).

### Brain tissue imaging

An Ultraflex III MALDI-TOF-TOF tandem mass spectrometer (Bruker Daltonics Inc., Bremen, Germany) was used for explorative imaging at low resolution. Briefly, mass spectra acquired for a *m/z* 140–1300 range were averaged from 300 consecutive laser shots. The raster spatial resolution was 90 μm. Depending on the image size, acquisition took 3 h. MALDI-MSI images was produced from the corresponding spectra to generate a relative intensity ion map at a given *m/z* value, using FlexImaging 4.1 software (Bruker Daltonics Inc.).

High resolution complementary analysis was performed using a high performance atmospheric pressure imaging ion source named AP-SMALDI5 AF (TransMIT GmbH, Giessen, Germany) connected to an orbital trapping mass spectrometer (Q-Exactive Orbitrap, Thermo Fisher Scientific, Bremen, Germany)^38^. This latter was operated in negative ion mode at a mass resolution of 70,000 at *m/z* 100 over a mass range of *m/z* 190–2000. The ion source is equipped with a diode laser (Flare NX343, λ = 343 nm), operating at a repetition rate of 2 kHz with an energy of 200 µJ/pulse. The imaging data were acquired in high speed continuous mode with a pixel size of 30 µm and a speed rate of 3.7 pixels/s.

### Data analysis

For LC–MS–MS and MSI, lipids identification was realized on LipidMaps^39^ and METASPACE software^40,41^ was used for high resolution spectra, based on peak exact mass and isotope profiles. A list of 216 identified lipids with a FDR of at least 20% was built. Identified lipids were classified according to their class (phospholipids, ceramides or plasmogens). Additionally, we also characterized the number of unsaturation and carbon chain length for each lipid.

MALDI images were generated using MSi Reader software^42^.

Normalized negative ion images of lipids were generated by dividing the given lipid signal by 16:0-d30-18:1 PA (m/z 703.670 ± 0.003) signal.

Normalized Positive ion images of lipids were generated by dividing the given lipid signal by the 16:0-d30-18:1 PC ([M + H] + *m/z* 790.772 ± 0.003 or [M + K] + *m/z* 828.728 ± 0.003, depending of the adduct of given positive ion) signal (Supplementary Figs. S2–S7).

### MSI and LC–MS–MS validation

To validate obtained results, three brains/group were analysed in MSI and in LC/MS/MS with technical replicate.

For MSI, brain sections from obese mice and chow-fed controls were acquired side by side in the same image (control section in the top and obese section in the bottom). A serial section of these brains were reacquired to obtain duplicate for each section. Then the brain sections from three obese and three chow-fed mice and their replicate were scanned. Ultimately, both the obese group and the control group contained six acquisitions. For each lipid, we performed a normalized relative quantification in the different selected region of interest (ROI) (based on brain atlas) to obtain an average quantification from each ROI. Then, Log-ratios of obese vs control were calculated for each lipid, and a paired t-test was performed on n = 6 (3 biological riplicate × 2 technical riplicate). Therefore, only lipids showing a p-value < 0.05 where kept for data analysis and average of significantly different lipids log-ratios was calculated and used for interpretation.

### Principal component analysis (PCA) and statistical analysis

A PCA using XLSTAT software (Addinsoft 2020. XLSTAT statistical and data analysis solution. NEW York, USA)^43^ was conducted on MALDI and LC–MS–MS data from brains of chow-fed controls and HFD-fed obese mice, before and after weight loss. PCA was used here to identify hidden variables between spectra taken from the brain regions of interest (ROI). It is a statistical tool used to reduce multidimensional data sets to lower dimensions and to identify new, meaningful, hidden differences between clusters. The first step of analysis consisted in a series of pre-processing data on the loaded spectra: deconvolution on the mean spectrum to reduce the background noise and baseline subtraction, then signal intensity normalization on internal standard signal. The software selects the vectors (principal component (PC)) that account for the greatest variation between the groups. A "best fitting" vector can be defined as one that minimizes the average squared distance from a point to the line^44^.

Body weight and body composition data were analysed using Prism (version 8). Unpaired t-test were used for statistical analysis. P < 0.05 indicate significance (Suppl. Information).

## Supplementary Information

Supplementary Figures.
