# Supplementary material for: Mass spectrometry imaging of mice brain lipid profile changes over time under high fat diet
Source: Sci Rep. 2021 Oct 4;11:19664. doi: 10.1038/s41598-021-97201-x (PMC8490458; doi:10.1038/s41598-021-97201-x)

Mass spectrometry imaging of mice brain lipid profile changes over time under high fat diet

Gianluca Sighinolfi[1]; Samantha Clark[2,3]; Landry Blanc[1] ; Daniela Cota[2,3]* ; Boutayna Rhourri-Frih[1*#]

[1]CBMN, Bordeaux, France; [2]INSERM, Neurocentre Magendie, Physiopathologie de la Plasticité Neuronale, U1215, F-33000 Bordeaux, France; [3]University of Bordeaux, Neurocentre Magendie, Physiopathologie de la Plasticité Neuronale, U1215, F-33000 Bordeaux, France

*Equally contributed to the work.

[#]Correspondence to: Boutayna Rhourri-Frih: boutayna.frih@u-bordeaux.fr

Supplementary data

Experimental protocol flowchart:

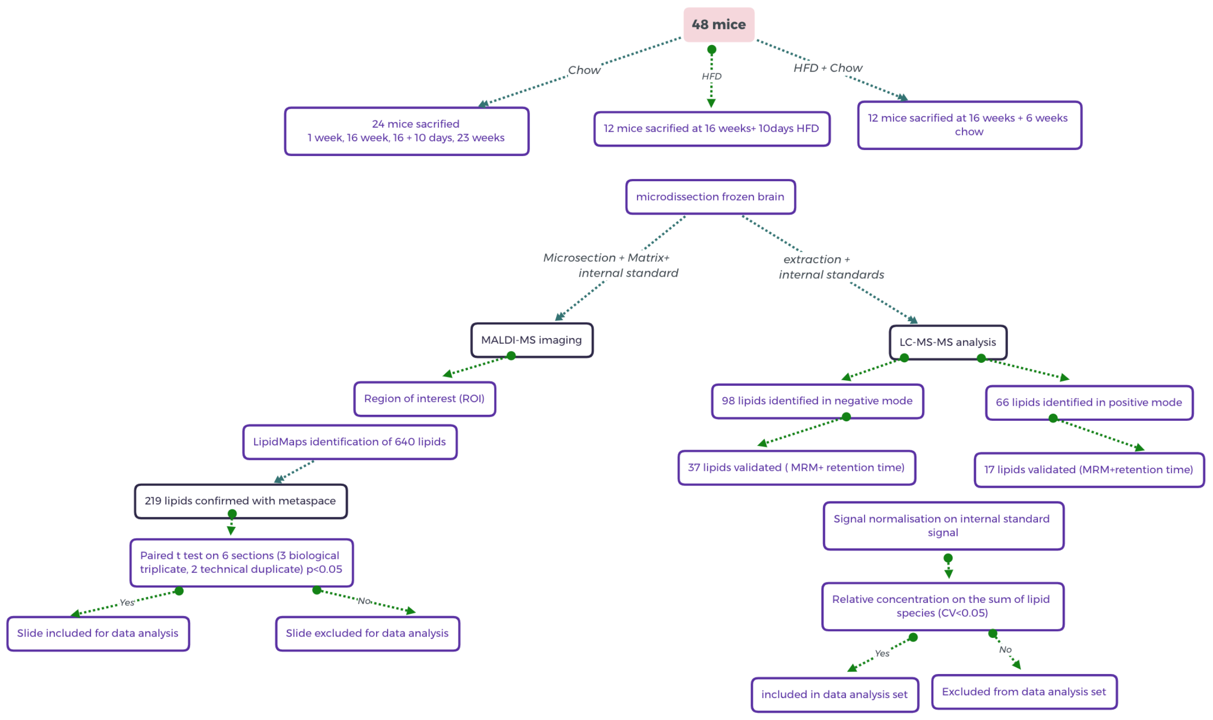

Figure S1: MALDI IMS images of persistent lipids (a) at 16weeks and (b) at 23 weeks

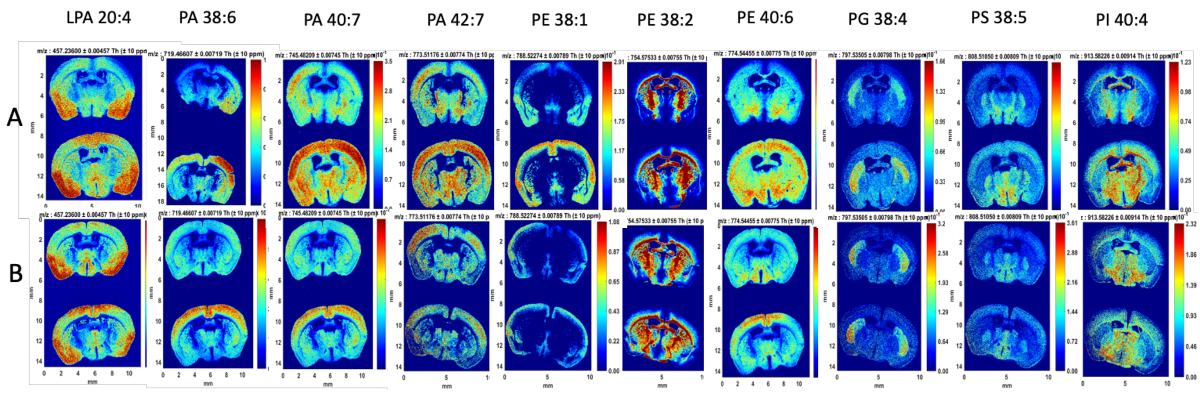

Figure S2: Multinomial logistic regression model examining the association between lipid species and the experimental groups (obese (T16), reversed diet group (T23)). Odds ratio in logarithmic scale represent the estimate changes in the concentration of the lipid species in obese and reversed diet group with respect to the reference group (Control) in the cortex-top region

Cortex Top

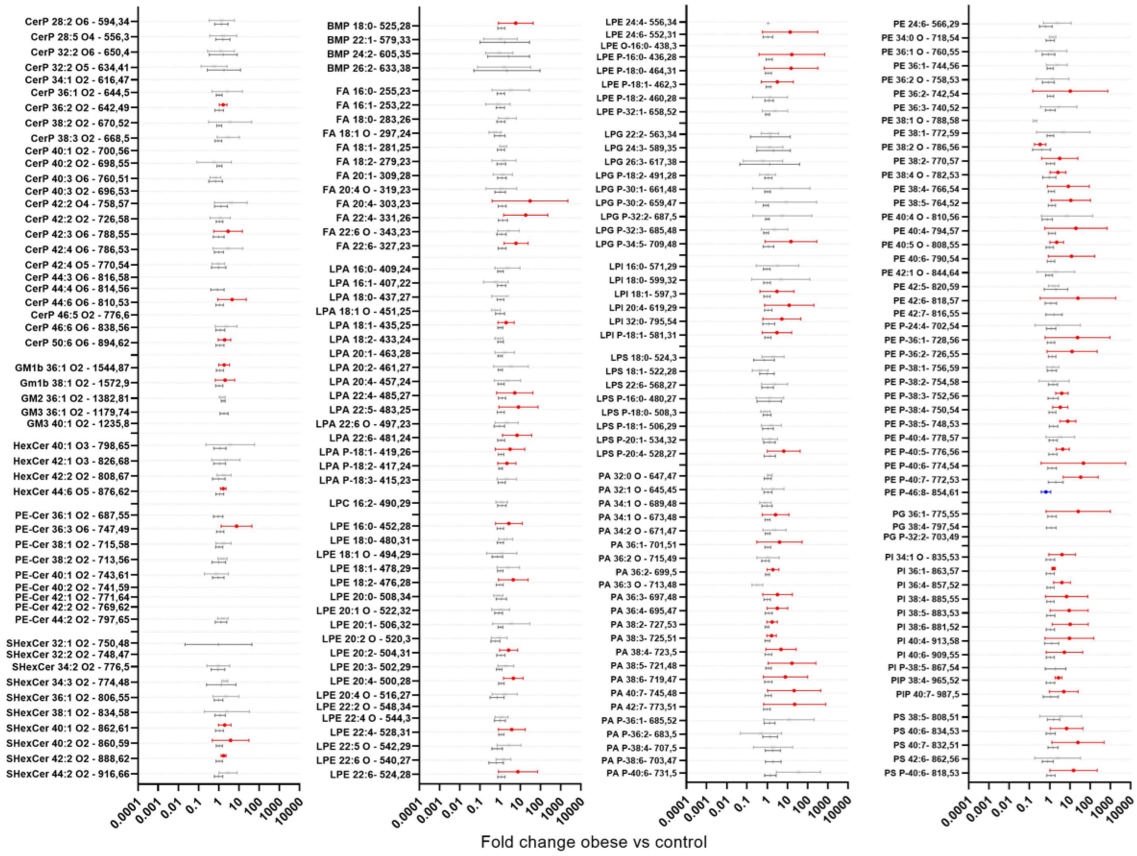

CerP 28:2 O6 - 594,34
CerP 28:5 O4 - 556,3
CerP 32:2 O6 - 650,4
CerP 32:2 O5 - 634,41
CerP 34:1 O2 - 616,47
CerP 36:1 O2 - 644,5
CerP 36:2 O2 - 642,49
CerP 38:2 O2 - 670,52
CerP 38:3 O2 - 668,5
CerP 40:1 O2 - 700,56
CerP 40:2 O2 - 698,55
CerP 40:3 O6 - 760,51
CerP 40:3 O2 - 696,53
CerP 42:2 O4 - 758,57
CerP 42:2 O2 - 726,58
CerP 42:3 O6 - 788,55
CerP 42:4 O6 - 786,53
CerP 42:4 O5 - 770,54
CerP 44:3 O6 - 816,58
CerP 44:4 O6 - 814,56
CerP 44:6 O6 - 810,53
CerP 46:5 O2 - 776,6
CerP 46:6 O6 - 838,56
CerP 50:6 O6 - 894,62
GM1b 36:1 O2 - 1544,87
Gm1b 38:1 O2 - 1572,9
GM2 36:1 O2 - 1382,81
GM3 36:1 O2 - 1179,74
GM3 40:1 O2 - 1235,8
HexCer 40:1 O3 - 798,65
HexCer 42:1 O3 - 826,68
HexCer 42:2 O2 - 808,67
HexCer 44:6 O5 - 876,62
PE-Cer 36:1 O2 - 687,55
PE-Cer 36:3 O6 - 747,49
PE-Cer 38:1 O2 - 715,58
PE-Cer 38:2 O2 - 713,56
PE-Cer 40:1 O2 - 743,61
PE-Cer 40:2 O2 - 741,59
PE-Cer 42:1 O2 - 771,64
PE-Cer 42:2 O2 - 769,62
PE-Cer 44:2 O2 - 797,65
SHexCer 32:1 O2 - 750,48
SHexCer 32:2 O2 - 748,47
SHexCer 34:2 O2 - 776,5
SHexCer 34:3 O2 - 774,48
SHexCer 36:1 O2 - 806,55
SHexCer 38:1 O2 - 834,58
SHexCer 40:1 O2 - 862,61
SHexCer 40:2 O2 - 860,59
SHexCer 42:2 O2 - 888,62
SHexCer 44:2 O2 - 916,66
BMP 18:0- 525,28
BMP 22:1- 579,33
BMP 24:2- 605,35
BMP 26:2- 633,38
FA 16:0- 255,23
FA 16:1- 253,22
FA 18:0- 283,26
FA 18:1 O - 297,24
FA 18:1- 281,25
FA 18:2- 279,23
FA 20:1- 309,28
FA 20:4 O - 319,23
FA 20:4- 303,23
FA 22:4- 331,26
FA 22:6 O - 343,23
FA 22:6- 327,23
LPA 16:0- 409,24
LPA 16:1- 407,22
LPA 18:0- 437,27
LPA 18:1 O - 451,25
LPA 18:1- 435,25
LPA 18:2- 433,24
LPA 20:1- 463,28
LPA 20:2- 461,27
LPA 20:4- 457,24
LPA 22:4- 485,27
LPA 22:5- 483,25
LPA 22:6 O - 497,23
LPA 22:6- 481,24
LPA P-18:1- 419,26
LPA P-18:2- 417,24
LPA P-18:3- 415,23
LPC 16:2- 490,29
LPE 16:0- 452,28
LPE 18:0- 480,31
LPE 18:1 O - 494,29
LPE 18:1- 478,29
LPE 18:2- 476,28
LPE 20:0- 508,34
LPE 20:1 O - 522,32
LPE 20:1- 506,32
LPE 20:2 O - 520,3
LPE 20:2- 504,31
LPE 20:3- 502,29
LPE 20:4- 500,28
LPE 20:4 O - 516,27
LPE 22:2 O - 548,34
LPE 22:4 O - 544,3
LPE 22:4- 528,31
LPE 22:5 O - 542,29
LPE 22:6 O - 540,27
LPE 22:6- 524,28
LPE 24:4- 556,34
LPE 24:6- 552,31
LPE O-16:0- 438,3
LPE P-16:0- 436,28
LPE P-18:0- 464,31
LPE P-18:1- 462,3
LPE P-18:2- 460,28
LPE P-32:1- 658,52
LPG 22:2- 563,34
LPG 24:3- 589,35
LPG 26:3- 617,38
LPG P-18:2- 491,28
LPG P-30:1- 661,48
LPG P-30:2- 659,47
LPG P-32:2- 687,5
LPG P-32:3- 685,48
LPG P-34:5- 709,48
LPI 16:0- 571,29
LPI 18:0- 599,32
LPI 18:1- 597,3
LPI 20:4- 619,29
LPI 32:0- 795,54
LPI P-18:1- 581,31
LPS 18:0- 524,3
LPS 18:1- 522,28
LPS 22:6- 568,27
LPS P-16:0- 480,27
LPS P-18:0- 508,3
LPS P-18:1- 506,29
LPS P-20:1- 534,32
LPS P-20:4- 528,27
PA 32:0 O - 647,47
PA 32:1 O - 645,45
PA 34:1 O - 689,48
PA 34:1 O - 673,48
PA 34:2 O - 671,47
PA 36:1- 701,51
PA 36:2 O - 715,49
PA 36:2- 699,5
PA 36:3 O - 713,48
PA 36:3- 697,48
PA 36:4- 695,47
PA 38:2- 727,53
PA 38:3- 725,51
PA 38:4- 723,5
PA 38:5- 721,48
PA 38:6- 719,47
PA 40:7- 745,48
PA 42:7- 773,51
PA P-36:1- 685,52
PA P-36:2- 683,5
PA P-38:4- 707,5
PA P-38:6- 703,47
PA P-40:6- 731,5
PE 24:6- 566,29
PE 34:0 O - 718,54
PE 36:1 O - 760,55
PE 36:1- 744,56
PE 36:2 O - 758,53
PE 36:2- 742,54
PE 36:3- 740,52
PE 38:1 O - 788,58
PE 38:1- 772,59
PE 38:2 O - 786,56
PE 38:2- 770,57
PE 38:4 O - 782,53
PE 38:4- 766,54
PE 38:5- 764,52
PE 40:4 O - 810,56
PE 40:4- 794,57
PE 40:5 O - 808,55
PE 40:6- 790,54
PE 42:1 O - 844,64
PE 42:5- 820,59
PE 42:6- 818,57
PE 42:7- 816,55
PE P-24:4- 702,54
PE P-36:1- 728,56
PE P-36:2- 726,55
PE P-38:1- 756,59
PE P-38:2- 754,58
PE P-38:3- 752,56
PE P-38:4- 750,54
PE P-38:5- 748,53
PE P-40:4- 778,57
PE P-40:5- 776,56
PE P-40:6- 774,54
PE P-40:7- 772,53
PE P-46:8- 854,61
PG 36:1- 775,55
PG 38:4- 797,54
PG P-32:2- 703,49
PI 34:1 O - 835,53
PI 36:1- 863,57
PI 36:4- 857,52
PI 38:4- 885,55
PI 38:5- 883,53
PI 38:6- 881,52
PI 40:4- 913,58
PI 40:6- 909,55
PI P-38:5- 867,54
PIP 38:4- 965,52
PIP 40:7- 987,5
PS 38:5- 808,51
PS 40:6- 834,53
PS 40:7- 832,51
PS 42:6- 862,56
PS P-40:6- 818,53
0.0001
0.001
0.01
0.1
1
10
100
1000
10000
Fold change obese vs control

Figure S3: Multinomial logistic regression model examining the association between lipid species and the experimental groups (obese (T16), reversed diet group (T23)). Odds ratio in logarithmic scale represent the estimate changes in the concentration of the lipid species in obese and reversed diet group with respect to the reference group (Control) in the cortex-bottom region

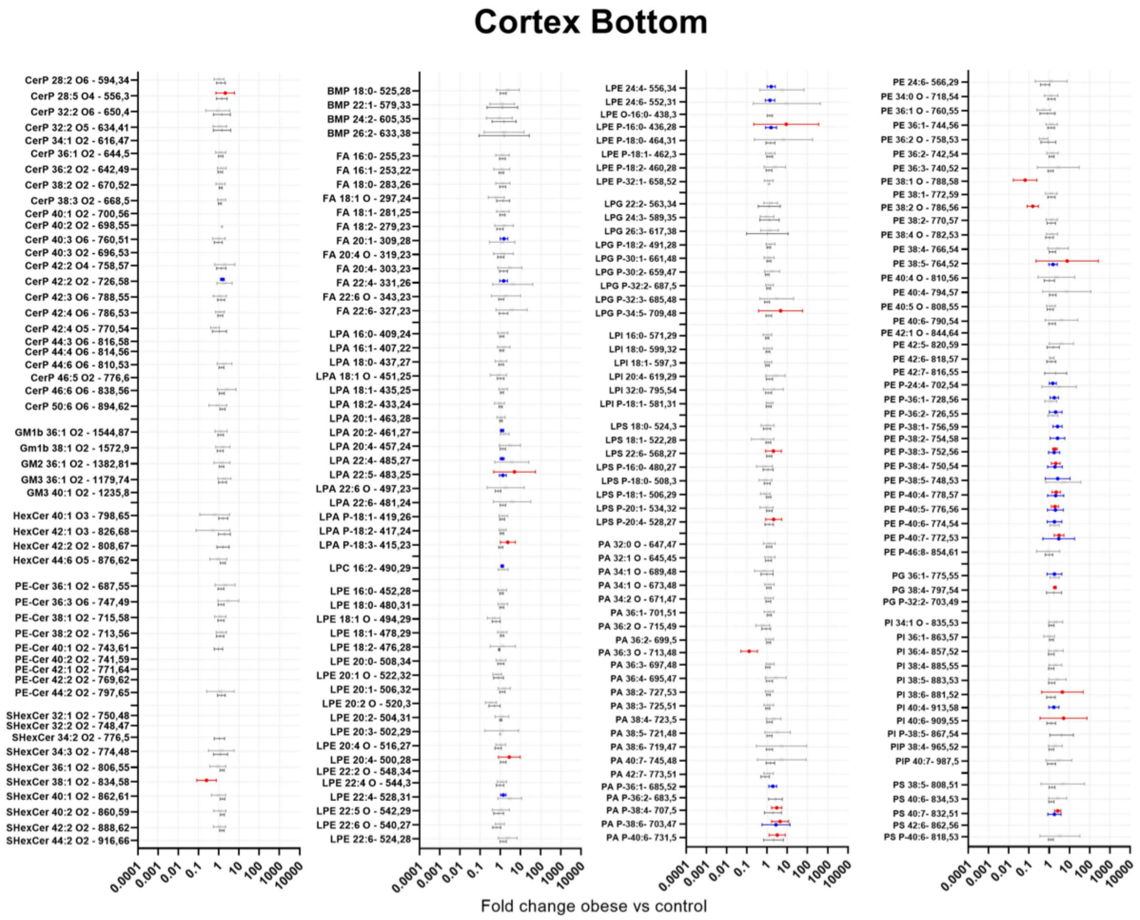

Figure S4: Multinomial logistic regression model examining the association between lipid species and the experimental groups (obese (T16), reversed diet group (T23)). Odds ratio in logarithmic scale represent the estimate changes in the concentration of the lipid species in obese and reversed diet group with respect to the reference group (Control) in the striatum region

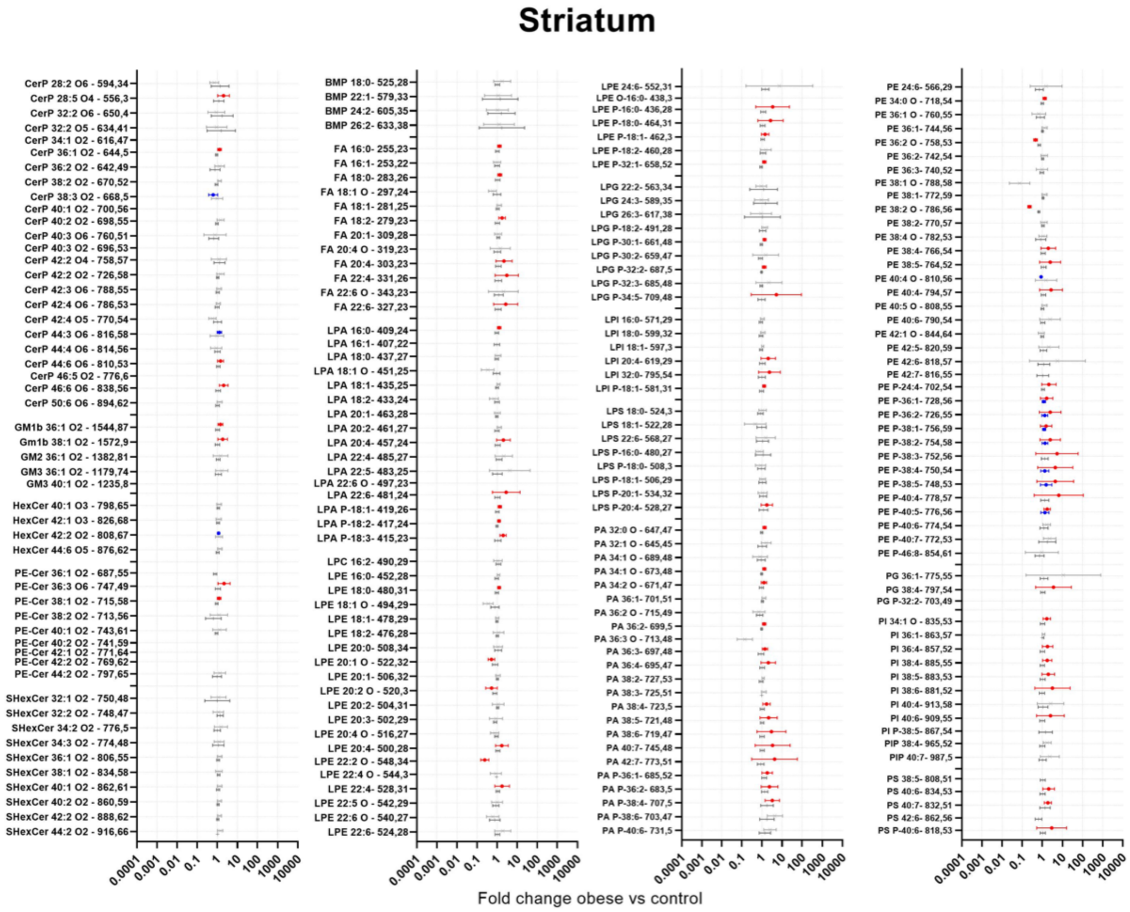

Figure S5: Multinomial logistic regression model examining the association between lipid species and the experimental groups (obese (T16), reversed diet group (T23)). Odds ratio in logarithmic scale represent the estimate changes in the concentration of the lipid species in obese and reversed diet group with respect to the reference group (Control) in the thalamic region

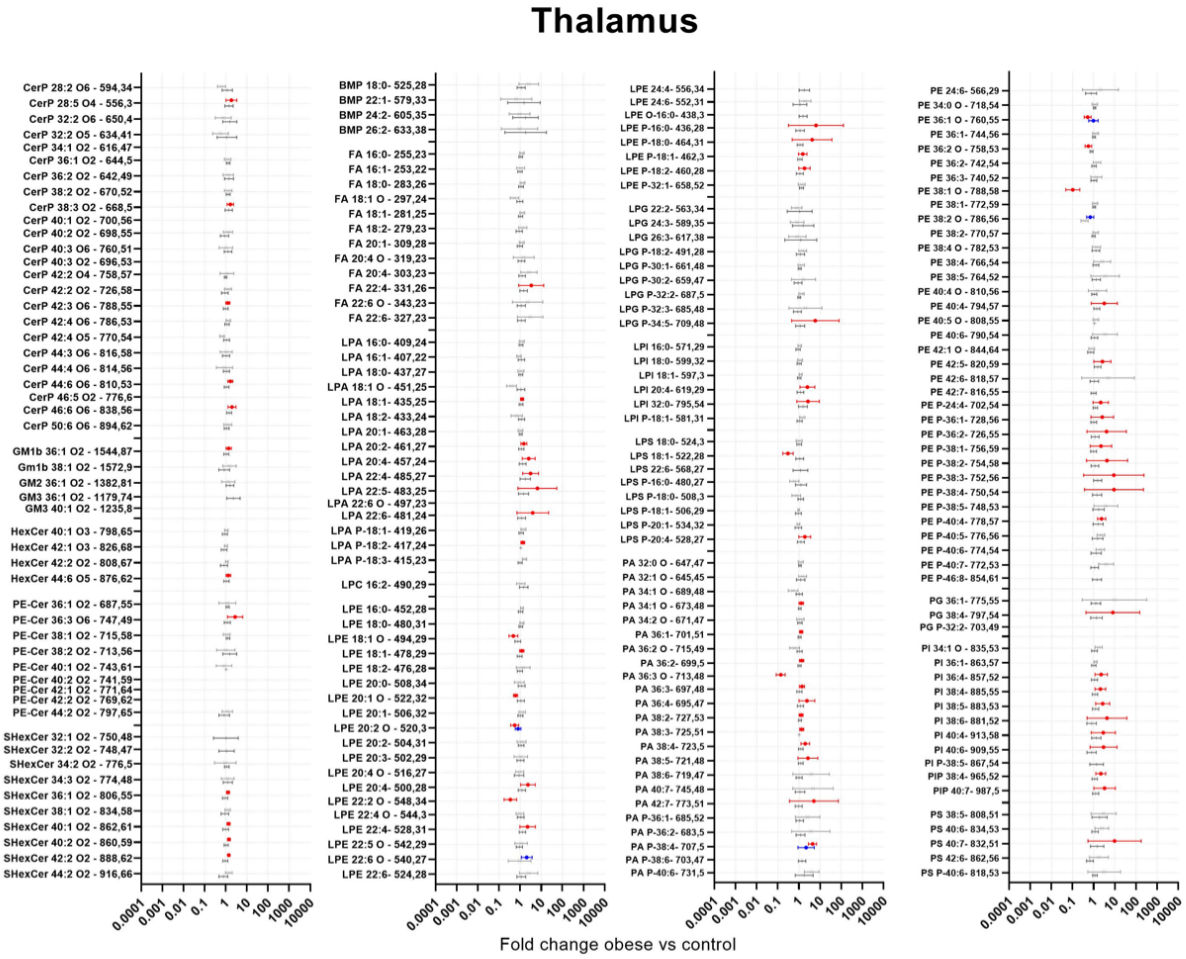

Figure S6: Multinomial logistic regression model examining the association between lipid species and the experimental groups (obese (T16), reversed diet group (T23)). Odds ratio in logarithmic scale represent the estimate changes in the concentration of the lipid species in obese and reversed diet group with respect to the reference group (Control) in the hypothalamic region

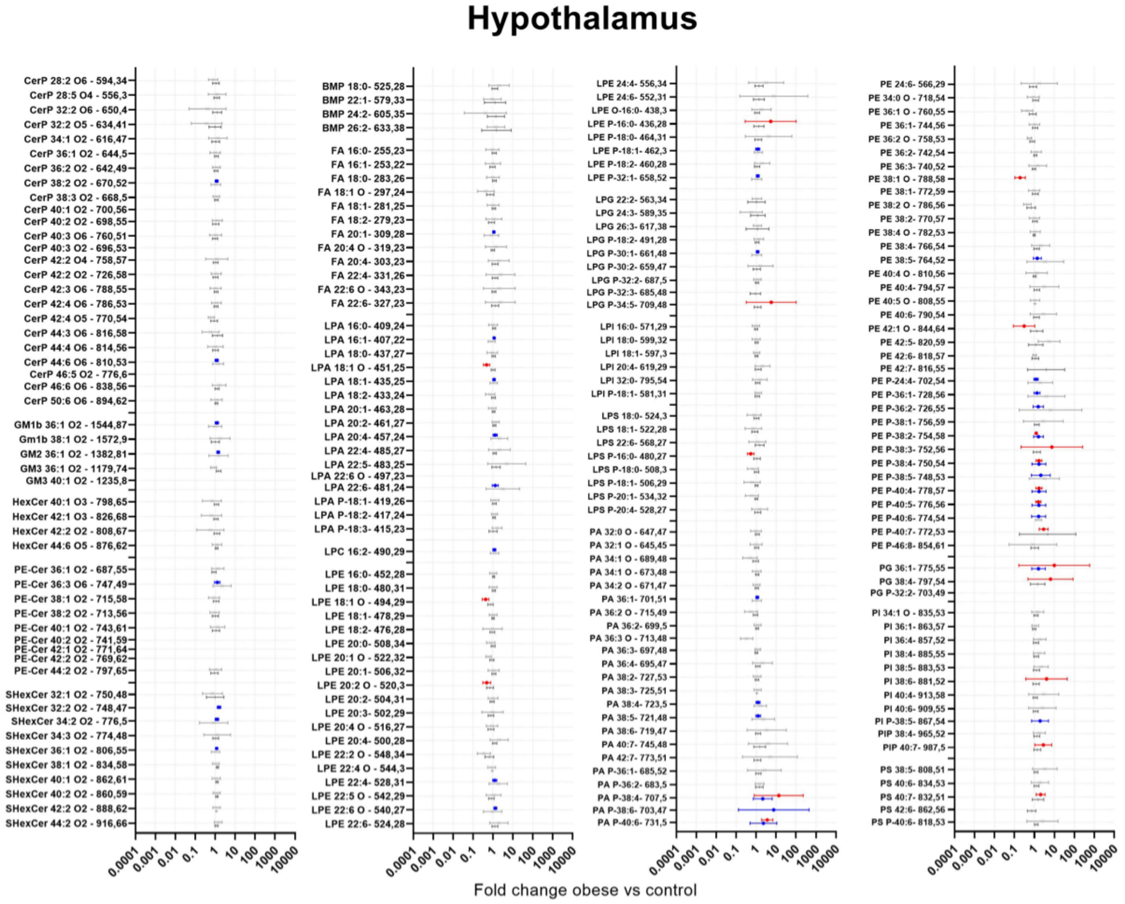

Figure S7: Multinomial logistic regression model examining the association between lipid species and the experimental groups (obese (T16), reversed diet group (T23)). Odds ratio in logarithmic scale represent the estimate changes in the concentration of the lipid species in obese and reversed diet group with respect to the reference group (Control) in the amygdala region

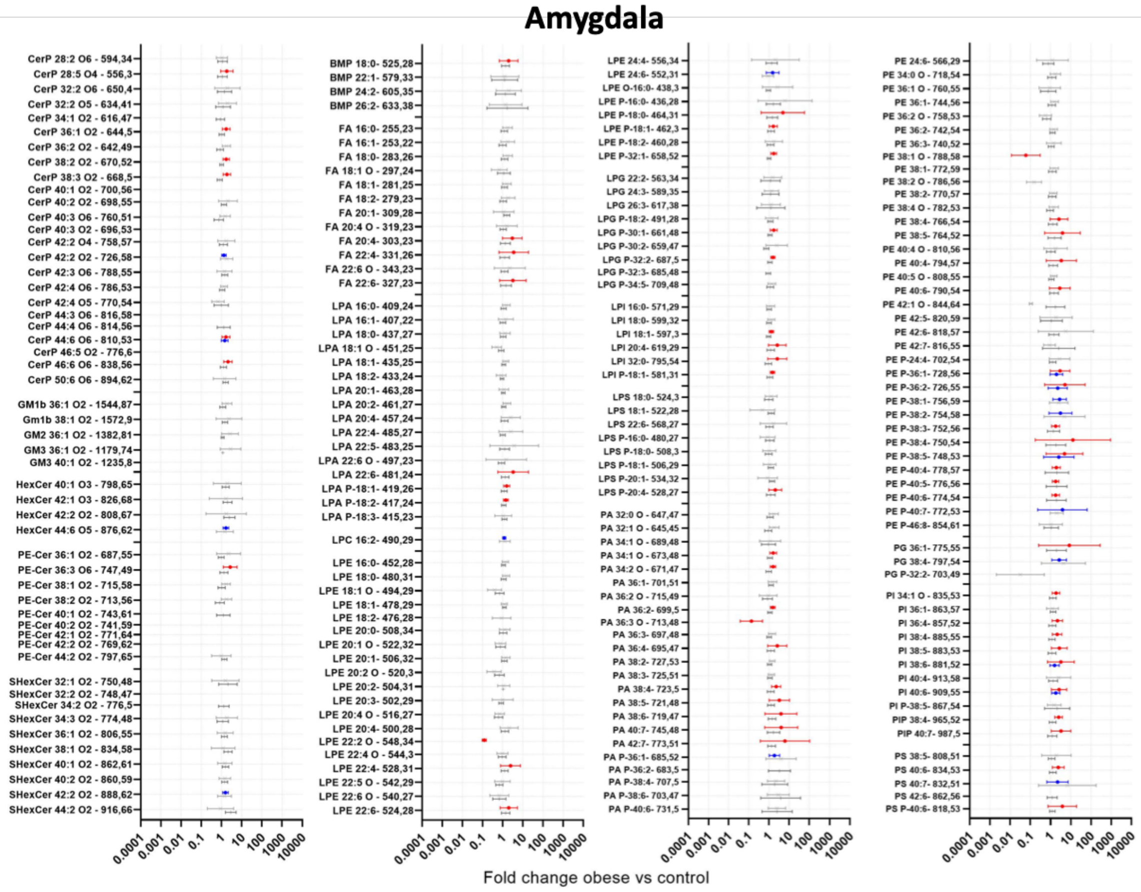

Supplement: Supplementary file 1 — Supplementary Figures. [file 41598_2021_97201_MOESM1_ESM.pdf]
